# Novel Types of Small RNA Exhibit Sequence- and Target-dependent Angiogenesis Suppression Without Activation of Toll-like Receptor 3 in an Age-related Macular Degeneration (AMD) Mouse Model

**DOI:** 10.1038/mtna.2015.34

**Published:** 2015-10-20

**Authors:** Masakatsu Takanashi, Katsuko Sudo, Shinobu Ueda, Shin-Ichiro Ohno, Yuko Yamada, Yasuhiro Osakabe, Hiroshi Goto, Yoshimichi Matsunaga, Akio Ishikawa, Yoshihiko Usui, Masahiko Kuroda

**Affiliations:** 1 Department of Molecular Pathology, Tokyo Medical University, Tokyo, Japan; 2 Animal Research Center, Tokyo Medical University, Tokyo, Japan; 3 Department of Ophthalmology, Tokyo Medical University, Tokyo, Japan

**Keywords:** innate immune, molecular target therapy, RNAi, TLR

## Abstract

RNA interference (RNAi) has become a powerful tool for suppressing gene expression *in vitro* and *in vivo*. A great deal of evidence has demonstrated the potential for the use of synthetic small interfering RNAs (siRNAs) as therapeutic agents. However, the application of siRNA to clinical medicine is still limited, mainly due to sequence-independent suppression of angiogenesis mediated by Toll-like receptor 3 (TLR3). Here, we describe novel types of synthetic RNA, named nkRNA and PnkRNA, that exhibit sequence-specific gene silencing through RNAi without activating TLRs or RIG-I–like receptor signaling. In addition, we confirmed the therapeutic effect for the novel types of RNA in an animal model of age-related macular degeneration (AMD) without retinal degeneration. These data indicate that nkRNA and PnkRNA are of great potential utility as therapies against blinding choroidal neovascularization due to AMD.

## Introduction

Age-related macular degeneration (AMD) is a complex disorder that primarily affects the macula, including the retinal pigment epithelium but also to a certain extent the photoreceptor layer and retinal neurons.^1,2^ The most common cause of vision loss from AMD is pathologic choroidal neovascularization (CNV), vascular leakage, and subsequent fluid accumulation in the neurosensory retina. Therefore, pharmacologic inhibition of vascular endothelial growth factor-A (VEGF-A) via targeted monoclonal antibody therapy has become the front line treatment for this blinding disease; this approach has high efficacy against AMD.^3,4^ Several other modalities of VEGF-A–targeted therapies have been investigated including posttranscriptional gene silencing via small interfering RNA (siRNA), an approach that showed great promise in preclinical studies. However, the clinical trials studying the first generation of siRNA-based drugs targeting VEGF-A for treatment of neovascular AMD were abruptly halted because they failed to reach the primary endpoints.^4^

Toll-like receptors (TLRs) belong to a family of innate immune receptors that detect and clear invading microbial pathogens. Specifically, intracellular TLRs such as TLR3, TLR7/8, and TLR9 recognize microbe-derived nucleic acids such as double-stranded RNA, single-stranded RNA, and CpG DNA, respectively.^5,6,7,8^ Many siRNAs with a wide range of targets, as well as siRNAs that are not competent for RNA interference (RNAi), suppress CNV in mice.^9,10^ Moreover, nontargeted (*i.e.*, not targeted against mammalian gene) and targeted siRNAs (against *Vegfa* or *Vegfr1*) suppressed CNV via TLR3, its adaptor TRIF, and induction of interferon-γ (IFNγ) and interleukin-12.^10^

To address some of these issues, we developed two novel types of synthetic RNA named nkRNA and PnkRNA.^11^ Both of these types of synthetic RNAs are single stranded and not chemically modified. Notably, nkRNA and PnkRNA exhibited sequence-specific gene silencing through RNAi. In addition, we transfected nkRNA and PnkRNA into murine endothelial cells and confirmed that they did not activate TLR3 signaling. Furthermore, we found that nkRNA and PnkRNA against VEGF suppressed CNV in laser-induced mouse models. These findings suggested that nkRNA and PnkRNA represent a suitable technology platform for molecularly targeted therapy.

## Results

### Novel types of RNAs induced downregulation of VEGF mRNA expression as effectively as siRNA

**Figure 1:**
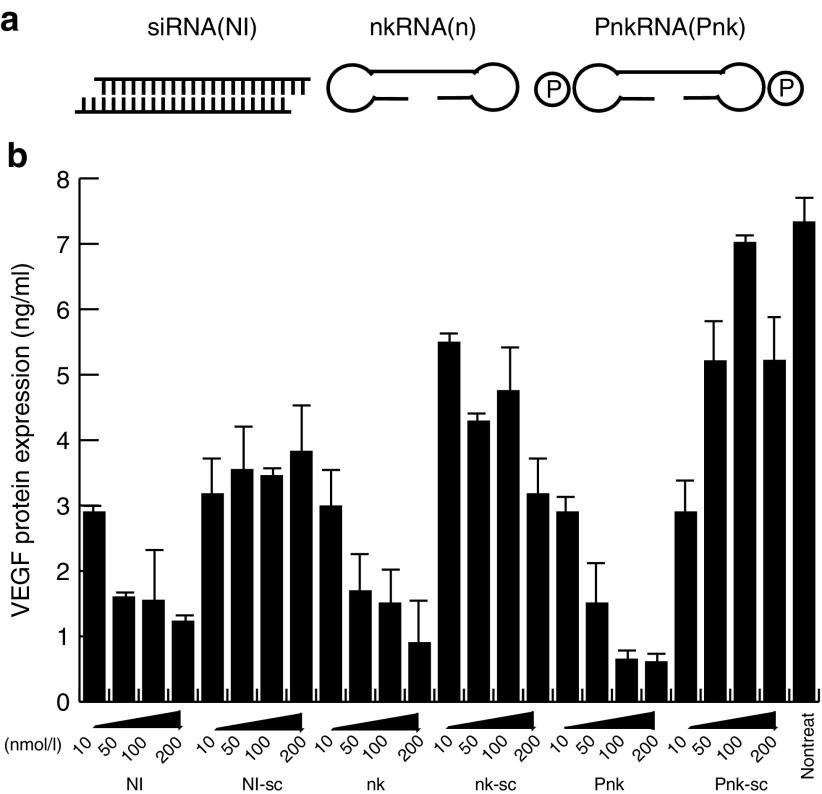
**Novel types of RNAs induced downregulation of VEGF mRNA expression as effectively as siRNA**. (**a**) Structure of novel RNA interference agents. Both nkRNA and PnkRNA were prepared as single-stranded RNA oligomers that underwent self-annealing, as shown. P indicates a proline derivative. (**b**) 10, 50, 100, and 200 µg of NIRNA, nkRNA, and PnkRNA against VEGF were transfected into mouse endothelial cells. Twenty-four hours after transfection, VEGF expression in the culture medium was measured using a VEGF ELISA kit. siRNA, small interfering RNA; VEGF, vascular endothelial growth factor.

To determine whether our new synthetic RNAs, nkRNA and PnkRNA, could induce knockdown of *VEGF* gene expression, we transfected these RNAs and conventional siRNA (NI) into the mouse endothelial cell line UVfemale2 and analyzed the expression of VEGF in the culture medium by ELISA. VEGF expression in the culture medium was reduced in a dose-dependent manner in cells transfected with all three types of RNAs. On the other hands, we observed no apparent knockdown of VEGF expression in cells transfected with scrambled RNAs (**Figure 1b**). These data indicate that nkRNA and PnkRNA can silence gene expression as efficiently as siRNA.

### nkRNA and PnkRNA induced TLR3 phosphorylation at low levels

**Figure 2:**
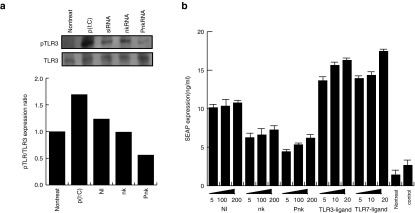
**nkRNA and PnkRNA induced TLR3 phosphorylation at low levels**. (**a**) Phosphorylated TLR3 was analyzed by western blotting. Ten micrograms of protein was loaded in each lane. The ratio of phosphorylated TLR3 to total TLR3 is shown in the graph. (**b**) NF-κB activity was measured using an NF-κB reporter in which SEAP cDNA was placed under the control of the κB response element. The numbers on x-axis show RNA concentrations (nM). SEAP, secreted alkaline phosphatase; siRNA, small interfering RNA; TLR, Toll-like receptor.

Double-stranded RNA, such as siRNA, is recognized by TLR3 and activates innate immunity via phosphorylation of TLR3 at tyrosine 759.^12,13^ To determine whether nkRNA and PnkRNA induce TLR3 phosphorylation, we transfected nkRNA, PnkRNA, NIRNA, or p(I: C) to mouse endothelial cells. Western blot analysis of lysates from cells 1 hour after transfection revealed that TLR3 was phosphorylated in cells that received p(I: C) or NIRNA. On the other hand, cells transfected with nkRNA or PnkRNA exhibited lower levels of TLR3 phosphorylation (**Figure 2a**), indicating that these novel RNAs do not activate TLR3. Phosphorylation of TLR3 leads to the activation of the NF-κB transcription factor.^14,15,16^ Activation of TLR3 by stimuli, such as viral dsRNA, leads to elevated expression of inflammatory cytokines, mediated by the NF- κB transcription factor. Therefore, to determine whether nkRNA and PnkRNA can induce NF-κB activation, we performed an NF-κB reporter assay using a vector encoding secreted alkaline phosphatase (SEAP) cDNA under the control of NF-κB response element. SEAP expression was lower in nkRNA- or PnkRNA-transfected cells than in NIRNA-transfected cells, and this reduction was dose dependent (**Figure 2b**). From these data, we concluded that nkRNA and PnkRNA were barely recognized by TLR3.

### nkRNA and PnkRNA did not induce an innate immune response, as demonstrated by expression of type I IFNs

**Figure 3:**
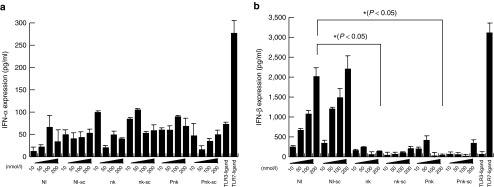
**nkRNA and PnkRNA did not induce an innate immune response, as demonstrated by expression of type I IFNs**. (**a,b**) Levels of IFNα (**a**) and IFNβ (**b**) from mouse endothelial cells in culture medium 24 hours after transfection of nkRNA, PnkRNA, NIRNA, or TLR ligands were measured by specific ELISA. The numbers in x-axis showed concentrations (nM) of RNAs. TLR3, TLR7, or TLR9 revealed the ligand against each receptor. IFN, interferon; TLR, Toll-like receptor.

The type I IFN pathway is typically associated with the innate immune response to the adaptors of TLR3.^17,18,19^ Hence, we analyzed type I IFN expression in nkRNA- and PnkRNA-transfected cells using the IFN ELISA kit. Although IFNα and IFNβ expression increased in a dose-dependent manner, there was no apparent difference between cells transfected with nkRNA, PnkRNA, and NIRNA (**Figure 3**). Expression of IFNβ was higher in cells transfected with NIRNA than in cells transfected with nkRNA or PnkRNA (**Figure 3**). According to these data, nkRNA and PnkRNA did not induce IFNβ expression. Therefore, we concluded that nkRNA and PnkRNA do not activate an innate immune response *in vitro*.

### nkRNA and PnkRNA did not induce expression of IFNβ in murine eyes

**Figure 4:**
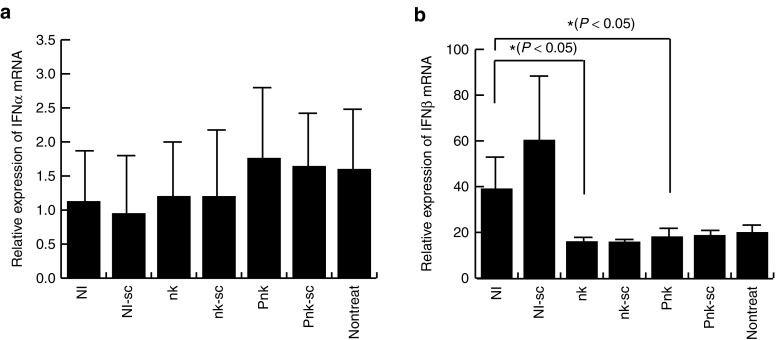
**nkRNA and PnkRNA did not induce expression of IFNβ in murine eyes**. To evaluate of activation for innate immune *in vivo* by nkRNA and PnkRNA, eyes of C57BL/6J mice were injected 1 µg of nkRNA, PnkRNA, or NIRNA. (**a**) IFNα and (**b**) IFNβ mRNAs from murine eyes 24 hours after administration mRNA were quantitated by real-time PCR. IFN, interferon.

Next, we asked whether administration of nkRNAs and PnkRNA into murine eyes would induce expression of type I IFNs. To address this question, we injected 1 µg of nkRNA, PnkRNA, or NIRNA into murine eyes. Twenty-four hours after injection, we excised the eyes, extracted RNA from them, and analyzed expression of IFNα and IFNβ by real-time quantitative PCR. IFNα was expressed at comparably low levels in all samples, whereas, IFNβ was expressed at lower levels in transfected with nkRNA and PnkRNA than in cells transfected with NIRNA (**Figure 4**). Thus, nkRNA and PnkRNA barely induced innate immunity in murine eyes.

### nkRNA and PnkRNA inhibited laser-induced CNV in a mouse model

**Figure 5:**
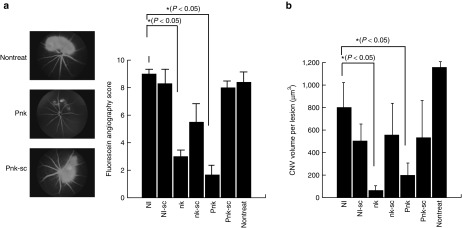
**nkRNA and PnkRNA inhibited laser-induced CNV in a mouse model**. (**a**) Fluorescein angiography. Neovascularization was evaluated based on leakage of blood fluid from neovasculature by fluorescein angiography. (**b**) Confocal micrograph of laser-induced CNV in mice. The retinas of eyes on RNA-treated mice after laser-induced coagulation were used to generate choroidal flatmounts, which were then examined with a scanning laser confocal microscope to visualize vessels. Vessels in the laser-treated area and superficial to this reference plane were judged as CNV. The area of CNV-related fluorescence was measured. CNV, choroidal neovascularization.

To determine whether the novel RNAs, which do not extensively activate innate immunity via TLR3, were able to reduce CNV in mice, we evaluated angiogenesis, as determined by leakage of blood, from neovascularization in response to laser coagulation (three shots per eye). The blood leakage scores in CNV mice were scored on a scale from 0 to 3; we estimated score for leakage of blood from vessels in eye. The scores were higher in mice that received NIRNA, nontreated, and scramble RNAs than in mice that received nkRNA or PnkRNA (**Figure 5a**).

We next examined the ability of nkRNA and PnkRNA to reduce angiogenesis by administering novel RNAs into the vitreous cavity of mice and comparing the CNV volume following laser coagulation 7 days after injection of RNAs. CNV volume was significantly reduced in eyes that received nkRNA and PnkRNA than in eyes that received NIRNA or scramble RNAs (**Figure 5b**). These data show that nkRNA and PnkRNA suppressed angiogenesis, indicating that the novel RNAs could be useful as therapies against CNV due to AMD.

## Discussion

siRNAs have attracted a great deal of attention as a new therapeutic platform for achieving target-specific gene silencing via double-stranded RNA (dsRNA)-mediated RNAi. However, the immune side effects due to TLRs activation are still problematic issues in the context of therapeutic applications. Recent work showed that siRNA activates TLR3, a long double-stranded viral RNA sensor, and activated TLR3 mediates target-independent angiogenesis suppression of CNV in mouse models.^10^

Recently, we developed a novel class of RNAi agents.^11^ Each of these agents is prepared as a single-stranded RNA that self-anneals into a unique structure containing a double-stranded RNA with an unpaired site, bound at the right and left ends by an oligonucleotide loop or a non-nucleotide molecule (proline derivative). Here, we showed that these novel RNAi therapeutic agents suppress expression of target genes without inducing type I IFN responses. In general, double-stranded RNAs are recognized by members of the membrane TLR and RIG-I–like receptor families (RIG-I, MDA5, and LGP2).^20,21^ When double-stranded RNA enters cells, both types of receptors activate NF-κB and/or IRF3/IRF7.^20,22,23,24,25^ In this study, we confirmed that nkRNA and PnkRNA induced low levels of TLR3 phosphorylation and NF-κB activation. In addition, we examined the type I IFN response. Together, these data demonstrated that nkRNA and PnkRNA can achieve sequence-specific gene silencing without activating the TLRs and RIG-I–like receptor.

How do nkRNA and PnkRNA evade the TLRs and RIG-I–like receptors? TLR3 recognizes double-stranded RNA, and recognition of ligands by TLR3 triggers signaling pathways that lead to the activation of transcription factors such as NF-κB, AP-1, and IRFs, which regulate the production of proinflammatory cytokines and type I IFNs.^23,26,27,28,29^ Because NIRNA is a double-stranded RNA, it is recognized by TLR3 and triggers the innate immune system. On the other hand, nkRNA and PnkRNA are prepared as single-stranded RNAs, but form double-stranded RNAs via self-annealing^11^ (**Figure 1a**). These double-stranded forms may enable the novel RNAs to escape recognition by TLR3. In addition, the ligand that activates TLR3 is double-stranded RNA or oligonucleotides of at least 40–50 base pairs in length.^30,31^ Although in this study we used oligonucleotides 41 (NIRNA), 62 (nkRNA), and 53 (PnkRNA) base pairs in length, only NIRNA activated TLR3. The structural conformation of the ligand of TLR3 plays an important role in recognition of TLR3.^6,32,33,34,35,36,37^ Furthermore, mutated virus with an altered conformation can escape the innate immune system, because such viruses are not recognized by TLRs.^38,39^ Because nkRNA and PnkRNA have loops at both ends, in between the sense and antisense nucleotide sequences, we concluded that the conformations of these unique RNA structures might be altered; therefore, these recognition of these RNAs by TLR3 was significantly impaired. PnkRNAs have prolines in both loops of the nucleotide sequence, and we predicted that the resultant alteration in structural conformation affected recognition of ligand by TLRs. The results of TLR3 phosphorylation indicated at low levels of the phosphorylation in the cell gene transferred with PnkRNA than that in the cell transferred with nkRNA. In addition, the data regarding NF-κB activation and IFNβ expression were consistent with the results described above.

The application of RNAi to clinical medicine is still limited, mainly due to activation of the innate immune system via TLRs. The VEGF silencing reagent causes side effects and exhibits sequence-independent suppression of angiogenesis.^10^

We confirmed the therapeutic effect of the novel RNAs in a mouse model of laser-induced CNV. nkRNA and PnkRNA were not associated with off-target expression of inflammatory cytokines in mice. *In vivo* data revealed that the novel RNAs, nkRNA and PnkRNA, repressed angiogenesis due to laser-induced coagulation. Thus, these novel RNAs may be effective as therapeutic reagents against CNV. Notably, escape from TLR3 recognition and VEGF mRNA silencing were better for PnkRNA than for nkRNA, indicating that is the superior therapeutic reagent. Accordingly, we concluded that nkRNA and PnkRNA may have great clinical utility as therapies against blinding CNV due to AMD. In particular, PnkRNA may become useful as a therapeutic agent against CNV.

## Materials and methods

*Cells.* The mouse endothelial cell line, UVfemale2,^40,41^ was purchased from the RIKEN cell bank and cultured in Dulbecco's Modified Eagle's medium (Life Technology, Carlsbad, CA) containing 10% fetal calf serum (FCS) penicillin and streptomycin. siRNA (NIRNA) or novel RNA oligonucleotides (nkRNA and PnkRNA) targeting mouse VEGF were transfected into endothelial cells using HiPerFect (Qiagen, Valencia, CA). One hour after transfection, cells were collected, and proteins were extracted for subsequent investigations.

*RNA interference.* siRNA (NI) and novel RNAs used in this study were as follows. Mouse VEGF (NI): sense 5′-ACCUCACCAAAGCCAGCACT-3′, antisense 5′- GUGCUGGCUUUGGUGAGGUTT-3′; mouse NI scramble: sense (NIsc) 5′- GCACAACACCCGCUCACAATT-3′, antisense 5′- UUGUGAGCGGGUGUUGUGCTT-3′; mouse VEGF nkRNA (nk): 5′- AACCUCACCAAAGCCAGCACUUCCCCACACCGGAAGUGCUGGCUUUGGUGAGGUUUCUUCGG-3′; mouse nk scramble RNA (nksc): 5′- AGCACAACACCCGCUCACAAUUCCCCACACCGGAAUUGUGAGCGGGUGUUGUGCUUCUUCGG-3′; mouse VEGF PnkRNA (Pnk): 5′-AACCUCACCAAAGCCAGCACUUCC-P5-GGAAGUGCUGGCUUUGGUGAGGUUUC-P5-G-3′; mouse Pnk scramble RNA (Pnksc): 5′- AGCACAACACCCGCUCACAAUUCC-P5-GGAAUUGUGAGCGGGUGUUGUGCUUC-P5-G-3′.

*Enzyme-linked immunosorbent assay.* IFN and VEGF protein levels were measured in culture medium of cells transfected with nkRNA, PnkRNA, or NIRNA, 24 hours after transfection, using cytokine-specific ELISA kits as follows: IFNα, VeriKine Interferon ELISA kit (PBL Biotechnology, Norwich, UK); IFNβ, EN424001 (Thermo Fisher Scientific, Waltham, MA); IFNγ, EM1011 (Thermo Fisher Scientific); and VEGF, Quantikine Mouse VEGF kit (R&D Systems, Minneapolis, MN).

*SEAPorter assay.* NF-κB activation by nkRNA, PnkRNA, or NIRNA transfection in cells was assayed using the SEAPorter assay kit (IMK-515; IMGENEX, San Diego, CA). Twenty-four hours after transfection, culture media from the oligo-transfected endothelial cells were analyzed.

*Real-time quantitative PCR.* RNAs were isolated from mouse eyes 24 hours after inoculation of 1 µg nkRNA, PnkRNA, or NIRNA per body using the Isogen reagent (Nippon Gene, Tokyo, Japan). Complementary DNA was synthesized using SuperScript II and Random Hexamers (Life Technology). Quantitative PCR (FastStart Universal SYBR Green Master; Roche, Basel, Switzerland) reactions were run on a Stratagene MX3000P thermocycler and analyzed using MxPro (Stratagene; Agilent Technologies, Santa Clara, CA). Glyceraldehyde 3-phosphate dehydrogenase mRNA was used as an internal control. IFN primers used in this study were as follows: IFNα (forward) 5′-TCTGATGCAGCAGGTGGG-3′, (reverse) 5′-AGGGCTCTCCAGACTTCTCGC-3′; IFNβ (forward) 5′-AGCACTGGGTGGAATGAGAC-3′, (reverse) 5′- TCCCACGTCAATCTTTCCTC-3′; IFNγ (forward) 5′- CACGGCACAGTCATTGAAAG-3′, (reverse) 5′-TTTTGCCAGTTCCTCCAGAT-3′; glyceraldehyde 3-phosphate dehydrogenase (forward) 5′-AAAATGGTGAAGGTCGGTGTG-3′, (reverse) 5′- TGGCAACAATCTCCACTTTG-3′.

*Western blot method.* Protein samples were suspended in sodium dodecyl sulfate loading buffer. After boiling, equal amounts (10 µg) of the proteins were run on 7.5% sodium dodecyl sulfate–polyacrylamide gel electrophoresis gels and then transferred to Immobilon membranes (Millipore, Bedford, MA) by semidry blotting. The membranes were probed with anti-TLR3 (Sc-86912; Santa Cruz Biotechnology, Dallas, TX) or anti-phospho-TLR3 (IMG-5348A; Imgenex/Novus Biologicals, Littleton, CO) antibodies using standard techniques. The signals were visualized using the ECL Plus Western Blotting Detection System (GE Healthcare, Piscataway, NJ) and detected on a LAS-3000 mini (Fujifilm, Tokyo, Japan).

*Animals.* All animal experiments were in accordance with the guide lines of Tokyo Medical University Institutional Animal Care and Use Committee. Female C57BL/6J mice (Japan SLC, Hamamatsu, Japan) between the age of 6 and 8 weeks were used to minimize variability. For all procedures, anesthesia was achieved by injection of under ketamine–xylazine anesthesia (120 and 6 mg/kg, respectively, i.p.).

*Induction of CNV.* Laser photocoagulation (532 nm, 200 mW, 100 ms, 75 µm, GYC-2000 Green/Dye; Nidek, Gamagori, Japan) was performed in three spots on one eye of each mouse. The laser spots were evaluated for the presence of CNV on day 7 by fluorescence angiography.

*Injection of siRNA or novel RNAs into mice.* On day 7 after laser photocoagulation, mice were anesthetized, and we administrated 1 µg of NIRNA, nkRNA, or PnkRNA into the vitreous cavity using a 32-gauge needle attached to a Hamilton syringe (Hamilton, Reno, NV).

*CNV volume.* Laser-induced CNV volume was measured by fluorescence angiography of Phoenix Micron III Retinal Imaging Microscope (Phoenix Research Lab, Pleasanton, CA) following i.p. injection of 0.1 ml of 1% FLUORESCITE (Alcon, Tokyo, Japan) into each mouse. CNV of retina was evaluated with fluorescence leakage of three categories (by grading score: I–III). Mice were anesthetized (ketamine/xylazine) and perfused through the heart with 1 ml of phosphate-buffered saline containing 50 µg fluorescein-labeled dextran (FITC-Dextran; Sigma-Aldrich, St. Louis, MO). The eyes were removed and fixed with 10% buffered formalin. The cornea and the lens were removed and the neurosensory retina was carefully dissected from the eyecup. Four relaxing radial incisions were made, and the remaining retinal pigment epithelium–choroid–sclera complex was flatmounted, Flatmounts were examined with a scanning laser confocal microscope (LMS710; Carl Zeiss, Oberkochen, Germany). Vessels were visualized by laser excitation and capture and capturing. About 1-µm step of horizontal optical sections were obtained from the surface of the retinal pigment epithelium–choroid–sclera complex. The area of CNV-related fluorescence was measured by computerized image analysis using the microscope software (ZEN; Carl Zeiss). The sum of the all area of CNV-related fluorescence in each horizontal section was used as an index of the CNV volume.

*Statistical analysis.* Statistical evaluation was performed on the basis of Student's *t*-test or Welch's *t*-test. *P* values less than 0.05 were considered significant.
